# Evaluation of comorbidity burden on disease progression and mortality in patients with interstitial pneumonia with autoimmune features: A retrospective cohort study

**DOI:** 10.1371/journal.pone.0316762

**Published:** 2025-02-04

**Authors:** Elena K. Joerns, Michelle A. Ghebranious, Traci N. Adams, Una E. Makris

**Affiliations:** 1 Mayo Clinic, Rochester, Minnesota, United States of America; 2 McGovern Medical School at University of Texas Houston, Houston, Texas, United States of America; 3 University of Texas Southwestern Medical Center, Dallas, Texas, United States of America; 4 Veterans Administration North Texas Health Care System, Dallas, Texas, United States of America; Ajou University School of Medicine and Graduate School of Medicine, REPUBLIC OF KOREA

## Abstract

**Background:**

Interstitial pneumonia with autoimmune features (IPAF) is a subset of interstitial lung disease that manifests with features of autoimmunity while not meeting classification criteria for a defined rheumatic disease. Comorbidity burden is an important prognostic indicator in various rheumatic and interstitial lung diseases, but few studies have commented on comorbidities in this population. This study was conducted to evaluate the association of individual comorbidities, the Charlson Comorbidity Index (CCI), and the Rheumatic Disease Comorbidity Index (RDCI) with lung disease progression and transplant/mortality outcomes in patients with IPAF.

**Methods:**

In a retrospective study, we evaluated the prevalence and severity of comorbidities in an institutional cohort of patients with IPAF. Using Cox regression, we correlated the association of individual comorbidities and comorbidity indices with time to lung disease progression (relative forced vital capacity decline of 10% or more) and with time to lung transplant/death. We compared the performance of CCI and RDCI in predicting outcomes.

**Results:**

History of cerebrovascular accident (CVA) or cardiovascular disease (CVD), moderate-severe chronic kidney disease, and fracture was associated with a faster onset of lung disease progression, while a history of gastroesophageal reflux was protective. History of CVA/CVD, diabetes mellitus, and lymphoma were associated with a faster onset of lung transplant/death. Both CCI and RDCI were associated with shorter time to lung disease progression and lung transplant/death in unadjusted analyses. However, only CCI was associated with shorter time to lung transplant/death in analyses adjusted for age, sex, pulmonary function, and radiographic pattern of lung lesion.

**Conclusions:**

CCI and RDCI may be useful tools in assessing prognosis in patients with IPAF in terms of both lung disease progression and mortality. Prospective studies are needed to further evaluate the performance of CCI and RDCI and the impact of optimizing comorbid conditions that may mitigate poor outcomes among patients with IPAF.

## Introduction

Interstitial pneumonia with autoimmune features (IPAF) is a subset of interstitial lung disease (ILD) that manifests with signs and symptoms of autoimmunity while not meeting classification criteria for a defined rheumatic disease (RD) [1]. Comorbidity burden is an important prognostic indicator in various RDs [2–5] and ILD [6, 7] and is associated with worse disease activity, physical function, quality of life, refractory treatment, and higher mortality risk [2, 8, 9], emphasizing the need for awareness, screening, and optimization of comorbid conditions. Multiple studies have examined the clinical characteristics and outcomes of IPAF patients [10–13], however, few studies have commented on the presence and prevalence of comorbidities in this population [14]. To our knowledge, no prior studies have evaluated the effect of comorbidities on lung function decline in IPAF.

Composite scoring systems or indices can be used to quantify the total burden of comorbid illnesses to measure the overall burden and impact of comorbidities more accurately and to assess prognosis [15]. Charlson Comorbidity Index (CCI), a summed score of 19 comorbidities weighted according to severity, was developed to predict one-year mortality in hospitalized patients. The CCI can predict various patient outcomes, including in-hospital mortality, length of hospital stays, readmission rates, functional decline, and healthcare utilization [9, 16]. CCI is also a strong prognostic predictor in ILD. A recent study demonstrated that the frequency of 3-year ILD-related events increased with increasing CCI; however, IPAF patients were not included in this cohort [16]. In addition, CCI does not account for common comorbidities seen in RDs such as hypertension, osteoporosis, obstructive sleep apnea, or depression which can significantly impact disease activity and quality of life [2, 9].

The rheumatic disease comorbidity index (RDCI) is comprised of 11 comorbid conditions and was developed based on self-report assessments from patients with RDs [15, 17]. It has performed well in predicting both physical disability and mortality in diseases such as rheumatoid arthritis and gout [15, 18]. However, its usefulness in presumably autoimmune ILD, such as IPAF, has not been evaluated.

The Gender-Age-Physiology (GAP) index was developed as a multidimensional prognostic staging system for ILD using four variables including gender (G), age (A), and two lung physiology variables (P)—forced vital capacity (FVC) and diffusing capacity for carbon monoxide (DLCO) [19]. Although initially validated in patients with idiopathic pulmonary fibrosis (IPF), the GAP model has also accurately predicted the risk of death in chronic non-IPF ILD [20–22]. A modified GAP index, known as the ILD-GAP index, includes a disease subtype variable that accounts for better-adjusted survival in ILD subtypes, including RD-associated ILD, chronic hypersensitivity pneumonitis, and idiopathic nonspecific interstitial pneumonia. The ILD-GAP model has predicted mortality in major chronic ILD subtypes across all stages of disease [21]. However, ILD-GAP does not consider the presence or severity of comorbidities, and a recent study demonstrated that the combination of ILD-GAP and CCI performs better in predicting outcomes in ILD than ILD-GAP or CCI alone [23].

Few studies have examined comorbidities [24–26] in IPAF and no prior studies have evaluated the association or effect of comorbidities, using indices such as the CCI or RDCI, with outcomes in IPAF. Given the inherent heterogeneity of IPAF and difficulty with prognostication for these patients, we aimed to improve the understanding of clinical course and prognosis in these patients. This study was conducted to determine the prevalence and type of comorbidities in an institutional cohort of patients with IPAF; to evaluate the effect of baseline comorbidities on lung disease progression and mortality; and to assess the performance of the CCI and RDCI, while adjusting for the ILD-GAP index, in predicting outcomes in IPAF patients.

## Materials and methods

### Cohort assembly

This single-center retrospective medical records review study was performed at the University of Texas Southwestern (UTSW) Medical Center. Patients seen in the UTSW Interstitial Lung Disease Clinic between January 2005 and August 2019 who met the European Respiratory Society (ERS)/American Thoracic Society (ATS) classification criteria for IPAF were included [1]. Data for this reasearch study was collected between 11/01/2021 and 8/31/2022 and the data abstractors had access to each participant’s individual level data. Date of entry into the cohort was considered as the first available pulmonary function test (PFT) date. The UTSW Medical Center Institutional Review Board approved the study prior to the initiation of data extraction (IRB #STU-2019-0913). Waiver of informed consent from University of Texas Southwestern Medical Center Institutional Review Board (IRB #STU-2019-0913) was obtained for each subject for this medical record review retrospective study. All aspects of the study were performed in accordance with all relevant guidelines and regulations including Declaration of Helsinki.

### Inclusion/exclusion criteria

All patients meeting 2015 ERS/ATS IPAF classification criteria were included for evaluation of prevalence of comorbidities and other baseline characteristics [1]. Patients were excluded from primary outcome evaluation if they did not have at least two sets of PFT data and excluded from secondary outcome evaluation if they had less than two visits recorded in the medical record.

### Baseline characteristics

Demographic data included age at ILD diagnosis, sex, race, ethnicity, and smoking status. ILD diagnosis date was the time point at which ILD was first observed on imaging. Sex, race, and ethnicity data was recorded as documented in the electronic medical record (EMR). Smoking status was assigned as smoker if the patient ever smoked and otherwise as never smoker. Medication data including type of medication and start and stop dates, was extracted from medical record.

Serological and clinical characteristics were recorded as documented in the EMR. Antibody presence was documented if present within the laboratory findings or if documented in the note by a physician. If the antibody was not checked, it was considered absent and was not counted toward meeting serological criteria of IPAF [1]. Clinical criteria was considered to be present if documented by the physician within the physical exam or history and absent if no documentation of the clinical characteristic was found within the EMR. Morphological features were assessed based on imaging findings as documented by radiologist and assessed by pulmonologist (TAN), and based on procedures including right heart catheterization and echocardiogram and documentation by the physician in the EMR.

Specific imaging features including lung lesion pattern, presence of honeycombing, emphysema, and air trapping, were assigned by a pulmonologist (TAN) based on high-resolution computed tomography scans (HRCT) of the chest. The presence of a usual interstitial pneumonia (UIP) pattern on imaging was documented, given the correlation of UIP with increased mortality and faster lung function decline in prior studies [11, 27].

### Comorbidities

Patient medical records were reviewed by an internal medicine physician (MAG) and rheumatologist (EKJ) using a standardized data extraction worksheet. Comorbidities were evaluated by searching for the key terms and reviewing pertinent documentation (including progress notes), laboratory values, and imaging at the time of entry into the cohort (defined as the date of the first PFTs recorded at the initial ILD clinic visit) (S1 File). Agreement between the reviewers was assessed after reviewing 50 charts to ensure accuracy.

### Calculation of comorbidity indices and ILD severity

CCI and RDCI were calculated according to standardized procedures and a data extraction worksheet (S1, S2 Tables) [15, 28].

The Interstitial lung Disease Gender-Age-Physiology (ILD-GAP) index (S3 Table) was calculated using five predictor variables (gender, age, FVC, DLCO, and ILD category) with assigned points to obtain a total score from 0 to 8 [21]. Since IPAF, by definition, is unclassified ILD due to lack of definable etiology such as RD [29], 0 points were assigned for the ILD category score for each patient in the cohort.

### Pulmonary function tests

PFT data included FVC, forced expiratory volume in one second (FEV1), FEV1/FVC ratio, and DLCO as percentages of predicted values. Baseline PFTs were the first available tests recorded at the first ILD clinic visit. All available additional PFT data were collected. If a patient underwent a lung transplantation, the last PFT data prior to transplant was included.

### Outcomes

The primary outcome was the time to lung disease progression, defined as relative %FVC decline of ≥10% after date of cohort entry. Secondary outcome was the time to lung transplant or all-cause mortality (whichever occurred first) from the time point of entry into the cohort. All outcomes were obtained from chart review.

### Statistical analysis

Prevalence of baseline characteristics and comorbidities in the IPAF cohort was expressed with descriptive statistics. Continuous and categorical variables were expressed as means with standard deviation and counts with percentages, respectively. We used Cox proportional hazards to estimate the hazard ratios (HRs) and 95% confidence intervals (CIs) for each outcome, first in an unadjusted model then adjusted for ILD-GAP and presence of UIP. All analyses were completed in Stata (V.17, College Station, TX).

## Results

### Baseline characteristics

The cohort was comprised of 201 patients meeting the 2015 ERS/ATS IPAF criteria, was predominantly female (76.1%), with a mean age at the time of initial PFTs of 61.8 ± 12.4 years, and an average time of follow-up of 5.6 ± 3.7 years. Baseline demographic and clinical characteristics are shown in Table 1. The prevalence of features included within the IPAF classification are shown in Table 2. One hundred and eighty-six (92.5%) of patients fulfilled serological domain, 98 (48%) fulfilled clinical domain, and 190 (94.5%) fulfilled morphological domain. The most common serological features were positive ANA and positive SSA, while most common clinical features were inflammatory arthritis and Raynaud’s phenomenon. Majority of the patients (76.1%) met morphological domain due to having non-UIP pattern on imaging. One hundred and seventy-six (88.0%) of patients were exposed to immunosuppressive or antifibrotic medications during their total follow-up time (Table 1). Most common immunosuppressants included prednisone, azathioprine, mycophenolate mofetil, and rituximab, while both nintedanib and pirfenidone were used as antifibrotic treatments in our cohort.

**Table 1:** Baseline features of the IPAF cohort.

Baseline characteristic	N = 201
ILD-GAP; Mean [SD]	3.2 [1.7]
CCI; Mean [SD]	3.2 [2.4]
RDCI; Mean [SD]	3.7 [1.3]
Age at initial PFTs, years; Mean [SD]	61.8 [12.4]
Male sex, n (%)	48 (23.9)
Race, n (%)	
White	140 (69.6)
Black	34 (16.9)
Asian	10 (5)
Other	17 (8.5)
Ethnicity, n (%)	
Non-Hispanic	170 (84.6)
Hispanic	31 (15.4)
Never smoke, n (%)	114 (65.5)
Baseline FVC, %; Mean [SD]	64.7 [19.1]
Baseline DLCO (n = 200), %; Mean [SD]	45.8 [20.3]
HRCT pattern, n (%)	
Unable to determine	21 (10.5)
LIP	5 (2.5)
NSIP	117 (58.2)
NSIP/OP	9 (4.5)
OP	1 (0.5)
UIP	48 (23.9)
Unexplained air trapping on HRCT, n (%)	94 (46.8)
Honeycombing on HRCT, n (%)	43 (21.4)
Emphysema on HRCT, n(%)	12 (6.0)
Follow-up time, years; Mean [SD]	5.6 [3.7]
Treatment during follow-up time to primary outcome (n = 191), n (%)	140 (73.3)
Immunosuppression*, n (%)	135 (70.7)
Antifibrotic**, n (%)	21 (11.0)
Treatment during follow-up time to secondary outcome (n = 200), n (%)	176 (88.0)
Immunosuppression, n (%)	174 (87.0)
Antifibrotic**, n (%)	37 (18.5)
Mortality outcome, n (%)	
Loss to follow-up after one visit	1 (0.5)
Alive at the end of follow-up	137 (68.2)
Death due to all causes	48 (23.9)
Lung transplant	15 (7.5)
Time to FVC decline of 10% or more (n = 104), years; Mean [SD]	2.2 [2.0]
Time to death or lung transplant (n = 63), years; Mean [SD]	4.2 [2.8]

IPAF–interstitial pneumonia with autoimmune features; ILD-GAP–interstitial lung disease-Gender-Age-Physiology Index; SD–standard deviation; CCI–Charlson Comorbidity Index; RDCI–Rheumatic Disease Comorbidity Index; PFTs–pulmonary function tests; FVC–forced vital capacity; DLCO–diffusing capacity of lung for carbon monoxide; HRCT–high resolution computed tomography; LIP–lymphocytic interstitial pneumonia; NSIP–non-specific interstitial pneumonia OP–organizing pneumonia; UIP–usual interstitial pneumonia.

*Immunosuppressive treatment included prednisone, mycophenolate mofetil or mycophenolic acid, azathioprine, rituximab, tofacitinib, intravenous immunoglobulin, and/or tacrolimus.

**Antifibrotic treatment included nintedanib and/or pirfenidone

**Table 2:** IPAF classification features within the cohort.

IPAF classification feature	N = 201 (%)
**Serological domain met **	186 (92.5)
ANA	161 (80.0)
ANA positivity according to IPAF classification criteria	115 (57.2)
RF present	50 (24.9)
RF positivity according to criteria	21 (10.4)
CCP	29 (14.4)
dsDNA	10 (5.0)
SSA	58 (28.9)
SSB	6 (3.0)
Scl-70	3 (1.5)
RNP	7 (3.5)
Smith	2 (1.0)
Jo-1	3 (1.5)
PL-7	5 (2.5)
PL-12	5 (2.5)
OJ	1 (0.5)
PM-Scl	5 (2.5)
MDA-5	0 (0)
U2-RNP	2 (1.0)
Ku	3 (1.5)
SRP	1 (0.5)
Mi2	1 (0.5)
**Clinical domain met **	98 (48.8)
Mechanics hands	10 (5.0)
Digital tip ulcerations	1 (0.5)
Inflammatory arthritis	40 (20.0)
Palmar telangiectasias	4 (2.0)
Raynaud’s phenomenon	42 (20.9)
Digital edema	11 (5.5)
Rash on extensor surface	5 (2.5)
**Morphologic domain met **	190 (94.5)
PAH	30 (14.9)
Air trapping in non-smoker	76 (37.8)
Pleural involvement	43 (21.4)
Pericardial involvement	35 (17.4)
Non-UIP radiographic pattern	153 (76.1)

### Prevalence of comorbidities

**Fig 1:**
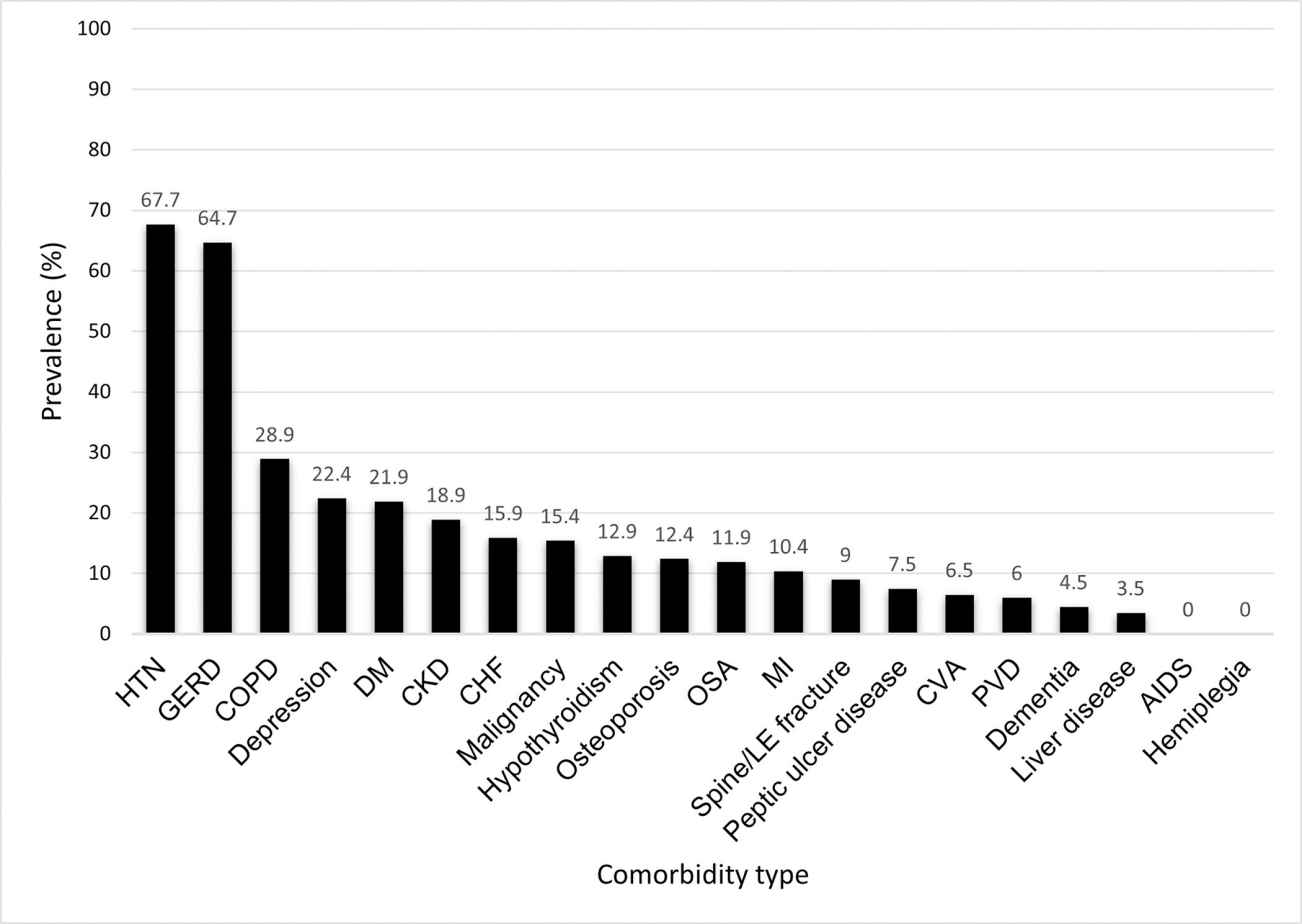
Comorbidity prevalence in the IPAF cohort. IPAF–interstitial pneumonia with autoimmune features; HTN–hypertension; GERD—gastroesophageal reflux disease; COPD—chronic obstructive pulmonary disease; DM—diabetes mellitus; CKD—chronic kidney disease; CHF—congestive heart failure; OSA—obstructive sleep apnea; MI—myocardial infarction; LE—lower extremity; CVA—cerebrovascular accident; PVD—peripheral vascular disease; AIDS—acquired immunodeficiency syndrome.

The prevalence of individual comorbidities is shown in Table 3. The most prevalent comorbidities in our cohort included hypertension (67.7%) and gastroesophageal reflux disease (GERD) (64.7%). More than 20% of the cohort had chronic obstructive pulmonary disease (COPD), depression, and diabetes mellitus (DM) (Fig 1). Approximately 15% of the cohort had a diagnosed malignancy, although no incidence of lung cancer was found. The average baseline CCI and RDCI were 3.2 (± 2.4) and 3.7 (± 1.3), respectively (Table 1).

**Table 3:** Baseline comorbidities of the IPAF cohort.

Baseline comorbidity	N = 201 n (%)
Myocardial infarction	21 (10.4)
Congestive heart failure	32 (15.9)
Peripheral vascular disease	12 (6)
Cerebrovascular accident*	13 (6.5)
Cardiovascular disease or cerebrovascular accident	41 (20.4)
Chronic obstructive pulmonary disease	58 (28.9)
Peptic ulcer disease	15 (7.5)
Liver disease	7 (3.5)
Diabetes mellitus	44 (21.9)
Diet-controlled	10 (5)
Uncomplicated requiring medications	25 (12.4)
With end-organ damage	9 (4.5)
Chronic kidney disease	
Any	38 (18.9)
Mild	34 (16.9)
Moderate-severe	4 (2)
Malignancy**	31 (15.4)
Solid tumor	29 (14.4)
Localized	25 (12.4)
Metastatic	4 (2)
Leukemia	0 (0)
Lymphoma	2 (1.0)
Acquired immunodeficiency syndrome	0 (0)
Hemiplegia	0 (0)
Fracture (hip/leg/spine)	18 (9)
Osteoporosis	25 (12.4)
Depression	45 (22.4)
Dementia	9 (4.5)
Hypothyroidism	26 (12.9)
Hypertension	136 (67.7)
Obstructive sleep apnea	24 (11.9)
Gastroesophageal reflux disease	130 (64.7)

IPAF–interstitial pneumonia with autoimmune features

*Including transient ischemic attack

**Including skin cancer and hematologic malignancy

### Primary outcome

**Fig 2:**
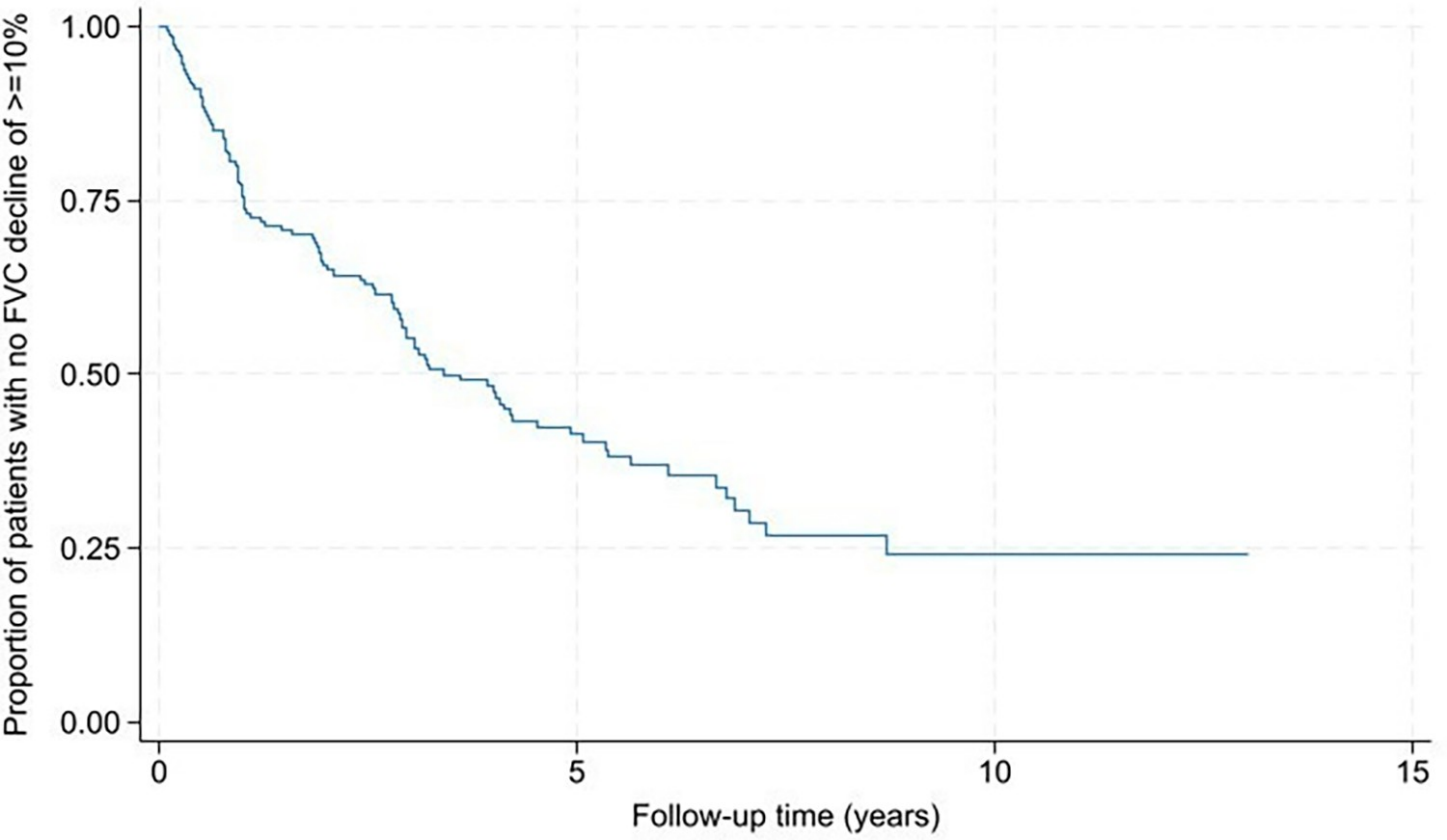
Lung function decline in the IPAF cohort. IPAF–interstitial pneumonia with autoimmune features; FVC–forced vital capacity.

**Fig 3:**
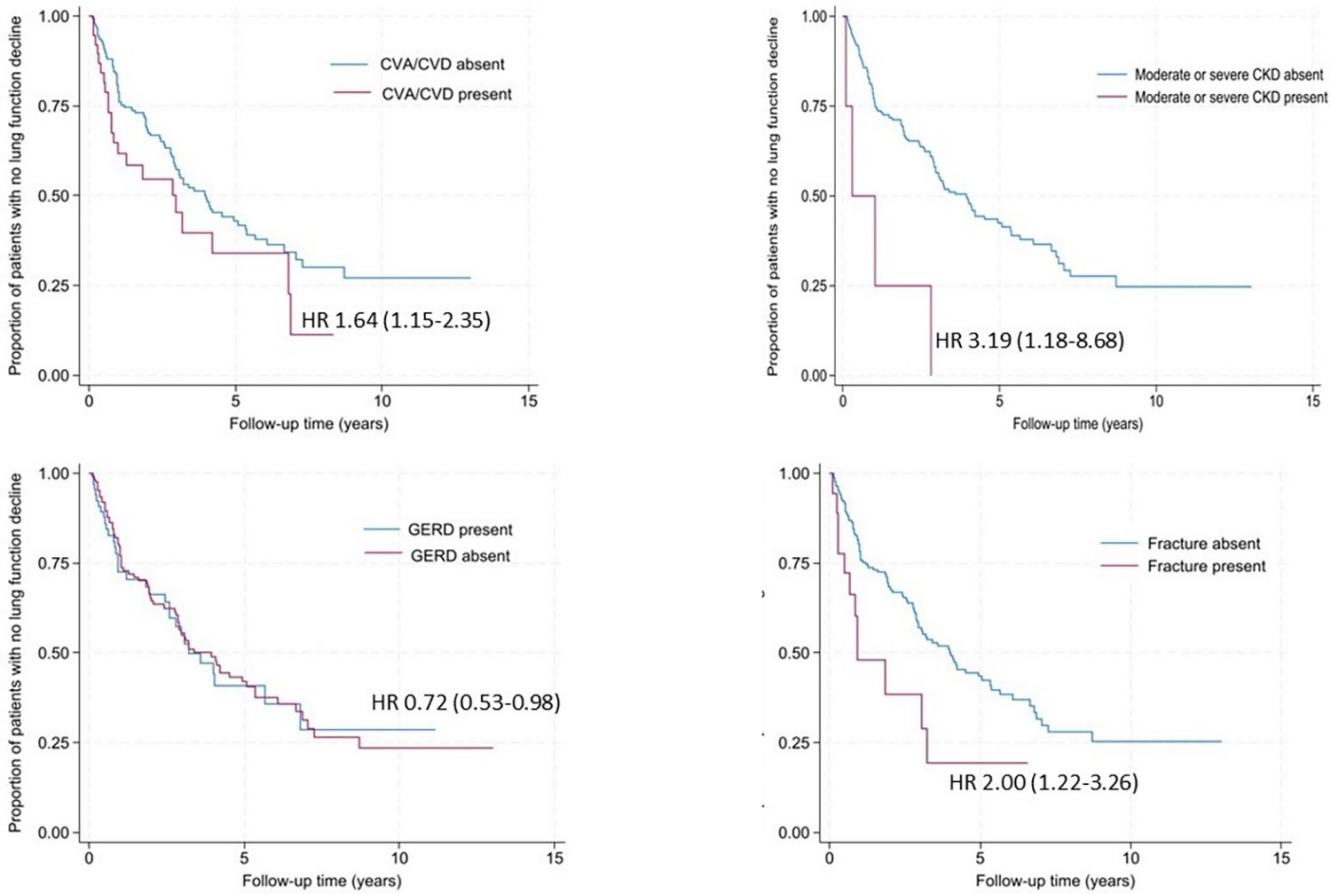
Comorbidities significantly associated with lung function decline in the IPAF cohort. IPAF–interstitial pneumonia with autoimmune features; CVA–cerebrovascular accident; CVD–cardiovascular disease; CKD—chronic kidney disease; GERD–gastroesophageal reflux disease; HR–hazard ratio.

One hundred and ninety-one (95.0%) patients had ≥2 PFTs and were included in lung function decline analysis. One hundred and forty (73.3%) patients were treated during their follow-up with immunosuppression and/or antifibrotic treatment (Table 1), with five patients receiving antifibrotic without immunosuppression and 119 patients receiving immunosuppression alone during follow-up. Of the 191 patients, 104 (54.5%) patients progressed over an average of 2.2 ± 2.0 years (Fig 2). In univariable analysis, non-Asian race, history of cerebrovascular accident (CVA) and/or cardiovascular disease (CVD), moderate to severe chronic kidney disease (CKD), and fracture were significantly associated with faster onset of lung disease progression (Fig 3). The presence of honeycombing and GERD were associated with delayed onset of lung disease progression. These associations remained significant when adjusted for ILD-GAP and UIP (Table 4).

**Table 4:** Association of baseline characteristics with time to lung function decline.

Baseline Characteristic	Univariable Analysis			Bivariable Analysis Model Adjusted for ILD-GAP			Bivariable Analysis Model Adjusted for UIP			Multivariable Analysis Model Adjusted for ILD-GAP and UIP		
N = 191	HR	95% CI	*P*	HR	95% CI	*P*	HR	95% CI	P	HR	95% CI	*P*
ILD-GAP	1.05	0.96–1.15	0.28	-	-	-	1.05	0.96–1.15	0.31	-	-	-
**CCI**	**1.11**	**1.04–1.19**	**0.001**	**1.11**	1.04–1.19	**0.002**	**1.11**	**1.03–1.18**	**0.003**	**1.10**	1.03–1.18	**0.01**
**RDCI**	**1.12**	**1.00–1.26**	**0.04**	1.12	0.999–1.25	0.05	**1.12**	**1.00–1.26**	**0.048**	1.12	0.996–1.25	0.06
Race												
White (referent)	1			1			1			1		
Black	0.71	0.48–1.04	0.08	0.71	0.49–1.05	0.08	0.71	0.49–1.05	0.08	0.72	0.49–1.05	0.09
**Asian**	**0.36**	**0.18–0.75**	**0.01**	**0.37**	**0.18–0.78**	**0.01**	**0.36**	**0.17–0.75**	**0.01**	**0.37**	**0.18–0.77**	**0.01**
Other	0.66	0.39–1.10	0.11	0.66	0.39–1.10	0.11	0.68	0.41–1.14	0.14	0.68	0.41–1.14	0.14
Hispanic ethnicity	1.02	0.68–1.52	0.93	1.00	0.67–1.49	>0.99	1.02	0.68–1.51	0.93	1.00	0.67–1.49	>0.99
Smoking history	1.10	0.82–1.49	0.52	1.08	0.80–1.46	0.63	1.06	0.78–1.44	0.73	1.03	0.76–1.41	0.84
UIP pattern on HRCT	1.28	0.92–1.79	0.14	1.27	0.91–1.78	0.16	-	-	-	-	-	-
Unexplained air trapping on HRCT	1.00	0.75–1.33	0.99	1.02	0.77–1.37	0.88	1.06	0.79–1.43	0.69	1.08	0.80–1.46	0.60
**Honeycombing on HRCT**	**0.64**	**0.45–0.92**	**0.02**	**0.65**	**0.45–0.93**	**0.02**	**0.66**	**0.46–0.95**	**0.03**	**0.67**	**0.46–0.97**	**0.03**
Emphysema on HRCT	1.14	0.57–2.27	0.71	1.16	0.58–2.35	0.67	1.09	0.55–2.20	0.80	1.13	0.55–2.30	0.74
Hypertension	1.12	0.83–1.51	0.47	1.11	0.82–1.51	0.49	1.12	0.82–1.51	0.48	1.11	0.82–1.50	0.51
Myocardial infarction	1.56	0.99–2.47	0.06	1.57	0.99–2.48	0.05	1.45	0.90–2.35	0.13	1.47	0.91–2.37	0.12
Congestive heart failure	1.15	0.77–1.71	0.49	1.12	0.75–1.68	0.56	1.15	0.77–1.72	0.48	1.13	0.76–1.68	0.56
Peripheral vascular disease	1.52	0.84–2.74	0.17	1.43	0.78–2.62	0.25	1.42	0.78–2.59	0.25	1.35	0.73–2.48	0.34
Cerebrovascular accident*	1.46	0.83–2.57	0.19	1.38	0.77–2.47	0.28	1.40	0.79–2.48	0.25	1.33	0.74–2.38	0.34
**Cardiovascular disease or cerebrovascular accident**	**1.64**	**1.15–2.35**	**0.01**	**1.61**	**1.12–2.31**	**0.01**	**1.60**	**1.11–2.29**	**0.01**	**1.56**	**1.09–2.25**	**0.02**
Chronic obstructive pulmonary disease	1.20	0.87–1.65	0.36	1.29	0.93–1.79	0.13	1.21	0.88–1.67	0.23	1.30	0.93–1.80	0.12
Obstructive sleep apnea	1.23	0.78–1.91	0.37	1.24	0.79–1.93	0.35	1.21	0.78–1.90	0.39	1.22	0.78–1.92	0.37
**Gastroesophageal reflux disease**	**0.72**	**0.53–0.98**	**0.04**	**0.70**	**0.52–0.96**	**0.02**	**0.71**	**0.52–0.96**	**0.03**	**0.69**	**0.50–0.93**	**0.02**
Peptic ulcer disease	0.89	0.52–1.55	0.69	0.91	0.52–1.57	0.73	0.89	0.52–1.55	0.69	0.90	0.52–1.56	0.72
Liver disease	1.03	0.45–2.32	0.95	1.07	0.47–2.43	0.87	0.97	0.43–2.20	0.94	1.00	0.44–2.28	>0.99
Diabetes mellitus (any)	1.10	0.78–1.55	0.58	1.09	0.77–1.54	0.62	1.11	0.79–1.57	0.54	1.11	0.78–1.56	0.57
None or diet controlled (referent)	1			1			1			1		
Uncomplicated requiring medications	0.84	0.55–1.29	0.42	0.83	0.54–1.28	0.41	0.86	0.56–1.34	0.51	0.86	0.56–1.33	0.50
With end organ damage	1.67	0.85–3.28	0.14	1.59	0.80–3.15	0.18	1.58	0.80–3.12	0.19	1.51	0.76–3.00	0.24
Chronic kidney disease (any)	1.43	0.98–2.07	0.06	1.40	0.96–2.04	0.07	1.40	0.97–2.04	0.075	1.38	0.95–2.01	0.09
None or mild (referent)	1			1			1			1		
**Moderate-severe**	**3.19**	**1.18–8.68**	**0.02**	**3.23**	**1.19–8.78**	**0.02**	**3.14**	**1.15–8.54**	**0.03**	**3.19**	**1.17–8.67**	**0.02**
Malignancy** (any)	1.01	0.68–1.51	0.95	1.00	0.67–1.49	>0.99	1.04	0.70–1.54	0.86	1.02	0.69–1.53	0.91
No solid tumor (referent)	1			1			1			1		
Local solid tumor	0.98	0.63–1.51	0.92	0.97	0.62–1.50	0.88	0.995	0.64–1.54	0.98	0.99	0.63–1.53	0.95
Metastatic solid tumor	1.89	0.70–5.14	0.21	1.86	0.68–5.06	0.22	1.88	0.69–5.12	0.21	1.87	0.69–5.07	0.22
Lymphoma	2.66	0.65–10.83	0.17	2.69	0.66–10.97	0.17	2.36	0.57–9.72	0.23	2.40	0.58–9.87	0.22
**Fracture (hip/leg/spine)**	**2.00**	**1.22–3.26**	**0.01**	**1.95**	**1.19–3.19**	**0.01**	**2.05**	**1.25–3.35**	**0.004**	**2.00**	**1.22–3.28**	**0.01**
Osteoporosis	1.15	0.75–1.77	0.53	1.14	0.74–1.76	0.54	1.09	0.71–1.69	0.69	1.09	0.70–1.69	0.70
Depression	1.21	0.85–1.71	0.28	1.21	0.85–1.71	0.28	1.20	0.85–1.70	0.29	1.20	0.85–1.70	0.29
Dementia	1.44	0.71–2.93	0.31	1.50	0.74–3.07	0.26	1.49	0.73–3.04	0.27	1.55	0.76–3.17	0.23
Hypothyroidism	1.24	0.80–1.92	0.34	1.24	0.80–1.92	0.34	1.29	0.83–2.02	0.26	1.29	0.83–2.01	0.26

RDCI–rheumatic disease comorbidity index; CCI–Charlson Comorbidity Index, ILD-GAP–Gender, Age, Physiology Index for Interstitial Lung Disease; PFTs–pulmonary function tests; FVC–forced vital capacity; DLCO–diffusing capacity of lung for carbon monoxide; UIP–usual interstitial pneumonia; HRCT–high resolution computed tomography; HR–hazard ratio; CI–confidence interval.

*Including transient ischemic attack

**Including skin cancer and hematologic malignancy

### Secondary outcome

**Fig 4:**
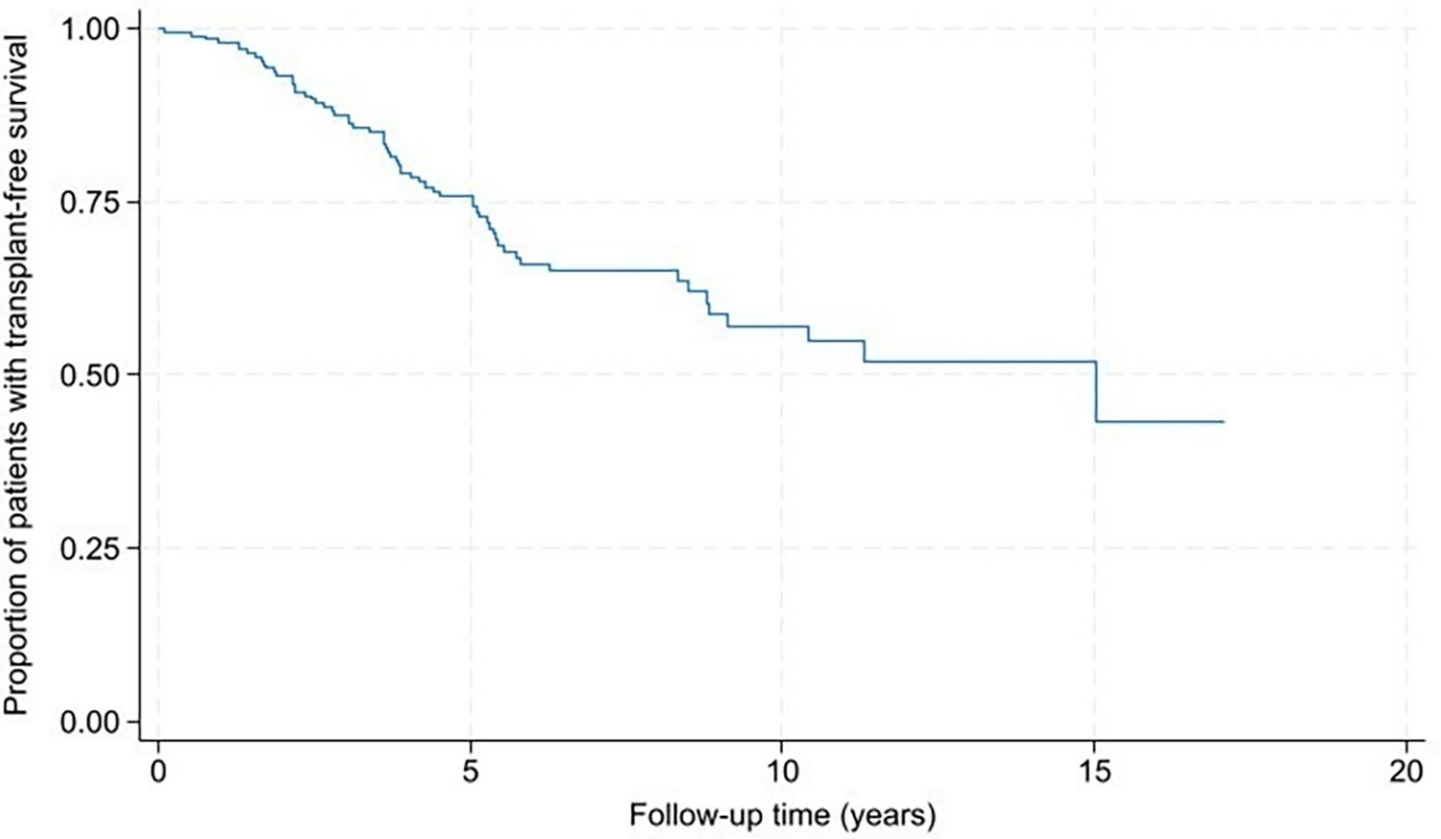
Mortality in the IPAF cohort. IPAF–interstitial pneumonia with autoimmune features.

**Fig 5:**
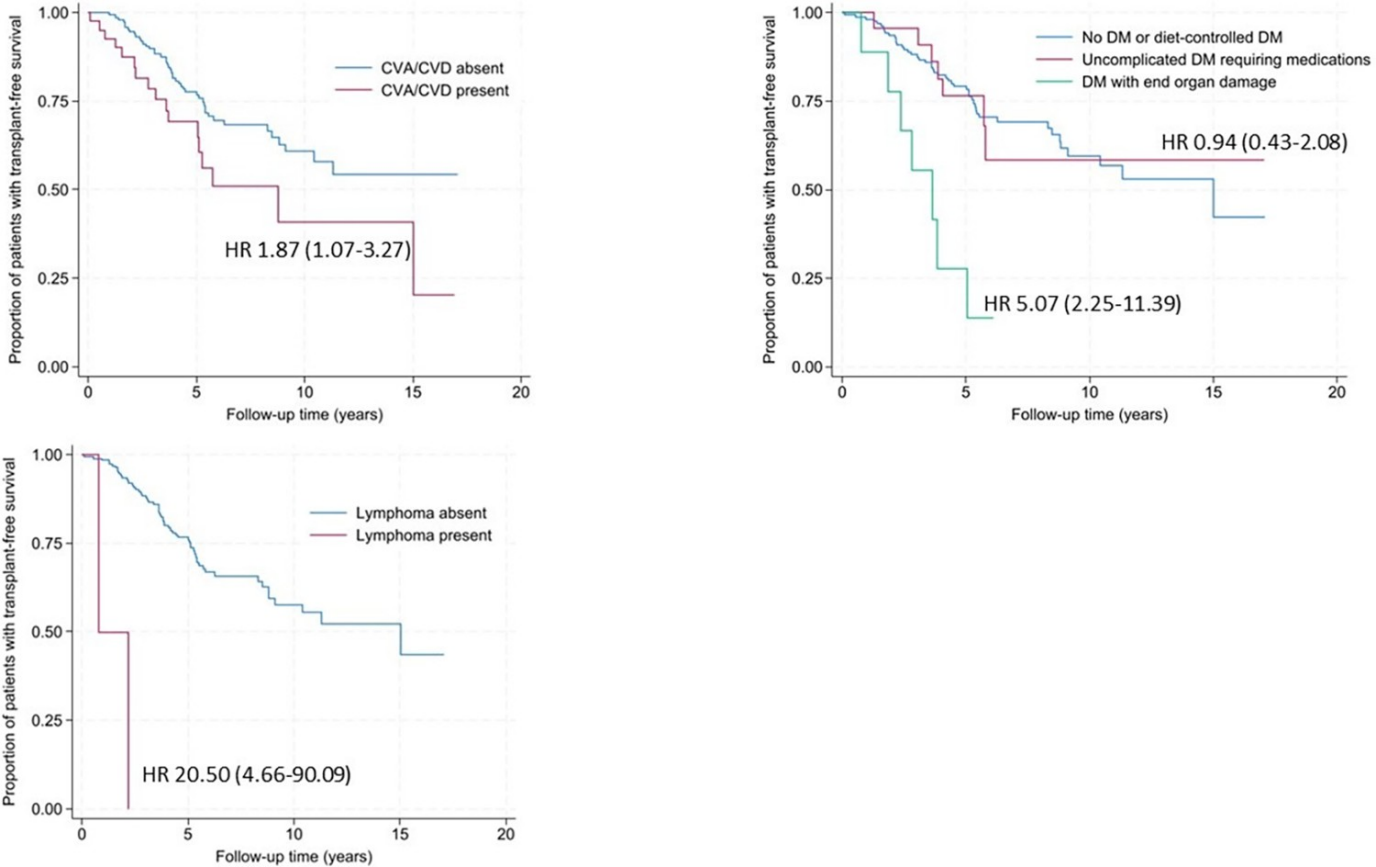
Comorbidities significantly associated with mortality in the IPAF cohort. IPAF–interstitial pneumonia with autoimmune features; CVA–cerebrovascular accident; CVD–cardiovascular disease; DM–diabetes mellitus; HR–hazard ratio.

Two hundred patients (99.5%) in our cohort had ≥1 medical visit recorded in the EMR. One patient was lost to follow-up after one visit. Mean duration to lung transplant or death was 4.2 +/-2.8 years (Fig 4). One hundred and seventy-six (88.0%) patients received treatment with immunosuppressive and/or antifibrotic medication during follow-up; only two patients received an antifibrotic without immunosuppression. Forty-eight (23.9%) patients died and 15 (7.5%) patients had lung transplant (Table 1). Smoking history, presence of UIP pattern, and history of CVA and/or CVD, DM (particularly with end-organ damage), and lymphoma were associated with significantly shorter time to lung transplant/death in univariable analysis (Fig 5). After adjusting for ILD-GAP, the UIP pattern on HRCT, DM, and lymphoma remained significantly associated with shorter time to lung transplant/death. After adjusting for the presence of UIP pattern, we found that smoking history, congestive heart failure (CHF), DM, and lymphoma were significant factors associated with the outcome. Interestingly, only DM with end organ damage and lymphoma remained significantly associated with lung transplant/death after adjusting for both ILD-GAP and UIP (Table 5).

**Table 5:** Association of baseline characteristics with time to lung transplant/death.

Baseline Characteristic	Univariable Analysis			Bivariable Analysis Model Adjusted for ILD-GAP			Bivariable Analysis Model Adjusted for UIP			Multivariable Analysis Model Adjusted for ILD-GAP and UIP		
N = 200	HR	95% CI	*P*	HR	95% CI	*P*	HR	95% CI	*P*	HR	95% CI	*P*
**ILD-GAP**	**1.53**	**1.31–1.80**	**<0.001**	-	-	-	**1.57**	**1.33–1.85**	**<0.001**	-	-	-
**CCI**	**1.18**	**1.07–1.30**	**0.001**	**1.13**	**1.02–1.26**	**0.02**	**1.16**	**1.04–1.29**	**0.01**	1.10	0.99–1.23	0.08
**RDCI**	**1.31**	**1.10–1.57**	**0.003**	**1.24**	**1.03–1.50**	**0.02**	**1.29**	**1.07–1.54**	**0.01**	1.20	0.99–1.46	0.06
Race												
White (referent)	1			1			1			1		
Black	0.55	0.27–1.12	0.10	0.63	0.30–1.29	0.20	0.59	0.29–1.21	0.15	0.68	0.33–1.41	0.30
Asian	0.17	0.026–1.35	0.10	0.26	0.04–1.92	0.19	0.19	0.03–1.38	0.10	0.28	0.04–2.08	0.21
Other	0.35	0.11–1.13	0.08	0.34	0.11–1.09	0.07	0.38	0.12–1.21	0.10	0.37	0.11–1.21	0.10
Hispanic ethnicity	1.43	0.79–2.60	0.24	1.18	0.64–2.17	0.60	1.40	0.77–2.53	0.27	1.19	0.65–2.19	0.57
**Smoking history**	**1.97**	**1.19–3.24**	**0.01**	1.42	0.84–2.42	0.19	**1.84**	**1.11–3.06**	**0.02**	1.32	0.77–2.24	0.31
**UIP pattern on HRCT**	**1.70**	**1.01–2.47**	**0.047**	**1.86**	**1.09–3.17**	**0.02**	-	-	-	-	-	-
Unexplained air trapping on HRCT	1.19	0.73–1.93	0.50	1.42	0.86–2.35	0.17	1.41	0.84–2.37	0.20	1.62	0.97–2.72	0.07
Honeycombing on HRCT	1.12	0.65–1.93	0.69	1.14	0.66–1.97	0.64	1.18	0.68–2.04	0.56	1.25	0.72–2.18	0.43
Emphysema on HRCT	0.50	0.16–1.59	0.24	0.70	0.22–2.26	0.56	0.45	0.14–1.43	0.18	0.65	0.20–2.08	0.46
Hypertension	1.33	0.77–2.29	0.31	1.18	0.68–2.05	0.56	1.39	0.80–2.42	0.24	1.19	0.68–2.07	0.54
Myocardial infarction	1.10	0.50–2.42	0.81	1.06	0.48–2.34	0.88	0.91	0.40–2.04	0.82	0.93	0.42–2.07	0.87
Congestive heart failure	1.83	0.97–3.45	0.06	1.34	0.70–2.55	0.37	**2.00**	**1.05–3.81**	**0.03**	1.47	0.77–2.82	0.24
Peripheral vascular disease	2.23	0.96–5.19	0.06	1.29	0.54–3.08	0.57	1.98	0.84–4.66	0.12	1.20	0.50–2.85	0.68
Cerebrovascular accident*	1.60	0.64–4.01	0.31	0.91	0.35–2.34	0.85	1.40	0.55–3.54	0.48	0.85	0.33–2.16	0.73
**Cardiovascular disease or cerebrovascular accident** *	**1.87**	**1.07–3.27**	**0.03**	1.36	0.76–2.43	0.29	1.72	**0.97–3.04**	0.06	1.27	0.71–2.25	0.42
Chronic obstructive pulmonary disease	0.86	0.48–1.51	0.59	1.08	0.61–1.92	0.79	0.87	0.49–1.53	0.62	1.06	0.60–1.88	0.83
Obstructive sleep apnea	1.24	0.59–2.61	0.57	1.59	0.75–3.38	0.23	1.23	0.59–2.59	0.58	1.61	0.75–3.44	0.22
Gastroesophageal reflux disease	0.78	0.46–1.31	0.34	0.76	0.45–1.28	0.30	0.73	0.43–1.24	0.24	0.69	0.40–1.17	0.17
Peptic ulcer disease	1.46	0.63–3.39	0.38	1.50	0.64–3.50	0.35	1.28	0.54–3.00	0.58	1.14	0.47–2.76	0.77
Liver disease	0.48	0.066–3.45	0.46	0.58	0.08–4.18	0.58	0.40	0.06–2.90	0.36	0.41	0.06–3.06	0.39
**Diabetes mellitus (any)**	**1.84**	**1.08–3.17**	**0.03**	**1.74**	**1.01–2.99**	**0.045**	**1.79**	**1.04–3.08**	**0.04**	1.71	0.99–2.94	0.05
None or diet controlled (referent)	1			1			1			1		
Uncomplicated requiring medications	0.94	0.43–2.08	0.88	0.94	0.42–2.08	0.88	0.95	0.43–2.10	0.90	0.98	0.44–2.18	0.96
**With end organ damage**	**5.07**	**2.25–11.39**	**<0.001**	**3.01**	**1.31–6.94**	**0.01**	**4.61**	**2.04–10.41**	**<0.001**	**2.81**	**1.23–6.41**	**0.01**
Chronic kidney disease (any)	1.37	0.76–2.49	0.30	0.96	0.52–1.78	0.89	1.25	0.68–2.28	0.47	0.88	0.48–1.63	0.68
None or mild (referent)	1			1			1			1		
Moderate-severe	0.82	0.11–5.91	0.84	0.75	0.10–5.44	0.78	0.69	0.10–5.00	0.71	0.71	0.10–5.14	0.74
Malignancy** (any)	1.44	0.77–2.71	0.26	1.18	0.63–2.24	0.60	1.42	0.75–2.66	0.28	1.20	0.64–2.27	0.57
No solid tumor (referent)	1			1			1			1		
Local solid tumor	1.43	0.72–2.81	0.31	1.11	0.56–2.22	0.76	1.46	0.74–2.88	0.27	1.14	0.57–2.26	0.71
Metastatic solid tumor	0.82	0.11–5.93	0.84	1.22	0.17–8.90	0.85	0.62	0.08–4.60	0.64	1.07	0.15–7.91	0.95
**Lymphoma**	**20.50**	**4.66–90.09**	**<0.001**	**27.77**	**6.23–123.66**	**<0.001**	**15.42**	**3.40–70.02**	**<0.001**	**20.30**	**4.44–92.8**	**<0.001**
Fracture	1.57	0.67–3.65	0.30	1.47	0.63–3.42	0.37	1.44	0.62–3.36	0.4	1.44	0.62–3.36	0.4
Osteoporosis	1.02	0.47–2.25	0.95	0.92	0.42–2.03	0.84	0.92	0.42–2.04	0.84	0.79	0.35–1.76	0.56
Depression	1.42	0.78–2.58	0.25	1.41	0.78–2.57	0.26	1.41	0.78–2.57	0.26	1.38	0.76–2.51	0.29
Dementia	1.48	0.46–4.74	0.51	1.97	0.61–6.34	0.26	1.71	0.53–5.52	0.37	2.33	0.71–7.59	0.16
Hypothyroidism	1.53	0.75–3.12	0.25	1.91	0.92–2.96	0.08	1.67	0.81–3.43	0.16	2.02	0.97–4.17	0.06

PFTs–pulmonary function tests; FVC–forced vital capacity; DLCO–diffusing capacity of lung for carbon monoxide; UIP–usual interstitial pneumonia; HRCT–high resolution computed tomography; HR–hazard ratio; CI–confidence interval; CCI–Charlson Comorbidity Index; RDCI–rheumatic disease comorbidity index; ILD-GAP–Gender, Age, Physiology Index for Interstitial Lung Disease.

*Including transient ischemic attack

**Including skin cancer and hematologic malignancy

### Effect of baseline comorbidity indices on outcomes

In univariable analysis, both CCI and RDCI were significantly associated with lung disease progression (Table 3) and lung transplant/mortality outcomes (Table 4). ILD-GAP was significantly associated with lung transplant/mortality but not with lung disease progression (Tables 3 and 4).

The association of CCI with lung disease progression, but not that of RDCI, remained significant when adjusted for ILD-GAP and ILD-GAP and UIP. When adjusted for the presence or absence of UIP pattern alone, both CCI and RDCI remained significant predictors of the outcome (Table 3).

Increasing CCI and RDCI were significantly associated with shorter time to lung transplant/death even when adjusted for ILD-GAP or UIP. However, neither CCI nor RDCI were significantly associated with the lung transplant/mortality outcome after adjusting for both ILD-GAP and UIP pattern in multivariable analysis. Higher ILD-GAP was associated with significantly shorter time to lung transplant/death outcomes in univariable analysis and when adjusted for UIP (Table 4).

## Discussion

In this study we explored the prevalence and the effect of baseline comorbidities on lung disease progression and lung transplant/mortality in a cohort of patients with IPAF. We also assessed the performance of two well-established comorbidity indices, CCI and RDCI, in predicting outcomes in this population. We found that comorbidity indices assist in prognostication of lung disease progression, but do not necessarily provide additional information to ILD-GAP when assessing lung transplant/mortality risk.

Hypertension and GERD were the most prevalent comorbidities in our cohort. This was consistent with a study by Oldham, et al., where GERD was the most prevalent (52.8%) comorbidity in an IPAF cohort raising suspicion for a potential contribution of GERD to disease pathogenesis in IPAF [11]. In addition, COPD, depression, and DM were found to be common comorbid conditions in our cohort, a finding which was not discussed in prior IPAF literature but has been demonstrated in other forms of ILD and IPF [7, 30–33]. Notably, none of the patients in our cohort had known lung cancer despite the increased risk of malignancy in patients with pulmonary fibrosis secondary to IPF [34, 35].

When evaluating the association of individual comorbidities on lung disease progression, this study revealed that multiple comorbidities such as a history of CVA/CVD, moderate/severe CKD and vertebral/lower extremity fracture, were associated with a faster onset of relative FVC decline of ≥10% or more from the time of cohort entry in patients with IPAF. Conversely, a history of GERD was associated with a longer time to lung disease progression.

Although it is difficult to ascertain the association between the various comorbidities, prior studies have commented on the effects of these comorbidities on lung disease progression in patients with other forms of ILD. For example, CVD is a treatable comorbidity frequently observed in IPF and has been linked to worse outcomes (including lung function decline), with proposed mechanisms including systemic inflammation, hypercoagulability, platelet activation, and oxidative stress [36–38]. A complex relationship between CKD and lung disease progression, particularly through alterations of fluid homeostasis and acid-base balance, has also been demonstrated [39, 40]. A potential explanation for the observed association of fracture and lung disease progression, in this study, is the increased prevalence of steroid use in patients with more severe disease which can thereby increase fracture risk. However, fracture was a baseline comorbidity in the cohort and thus steroid use would not be expected to contribute significantly to its prevalence at the time of baseline data collection. To evaluate this possibility, data regarding osteoporosis was also collected, but did not demonstrate a significant association with the studied outcomes. In other autoimmune diseases, such as rheumatoid arthritis, osteopenia and osteoporosis have correlated with higher mortality and fracture risk, when compared to the general population [17]. Interestingly, GERD was found to be a protective factor for lung disease progression in our cohort, a finding which varies from prior studies that considered GERD to be a risk factor for ILD development [41]. One consideration is the high prevalence of proton pump inhibitor use in patients with IPAF and GERD, an intervention hypothesized to slow lung disease progression in patients with IPF [42, 43]. Additionally, this finding may be representative of selection bias given that patients with GERD may suffer from bothersome symptoms including chronic cough which may result in seeking medical care sooner and may lead to earlier diagnosis and initiation of therapy. Based on our results, the association of CVD, CKD, fracture risk, and GERD with lung disease progression in patients with IPAF warrants further investigation.

Importantly, few prior studies have explored the prognostic impact of comorbid conditions in IPAF. Two studies suggested that hypothyroidism was associated with greater mortality [24, 25], but a diagnosis of obstructive sleep apnea (OSA) was correlated with better survival when compared to IPAF patients without these conditions [24]. Malignancy was noted to be a serious comorbidity associated with faster progression of fibrotic lung disease in RD-ILD and IPAF [26]. In our study, OSA and hypothyroidism were not significantly associated with outcomes, but lymphoma, CKD, and DM, were associated with shorter time to lung transplant/mortality.

We evaluated the performance of the CCI and RDCI in patients with IPAF and found that both CCI and RDCI were helpful in predicting lung function and transplant/mortality outcomes, even after adjusting for UIP pattern. However, the performance of indices was variable for both outcomes when adjusted for both ILD-GAP and UIP pattern. Notably, CVA/CVD and fracture, which are components of RDCI, but not CCI, were important comorbidities associated with poor outcomes in our study. Additionally, depression was highly prevalent in our cohort but is only reflected within the RDCI score. These findings suggest that both CCI and RDCI may be useful tools for prognosticating outcomes in IPAF patients.

Our study has several strengths. We had a large, diverse IPAF cohort and the ability to explore numerous comorbidities. Sixteen percent of our cohort included Black patients, increasing the external validity of our findings. Comorbidities have been variably explored in cohorts of patients with IPAF, resulting in highly heterogeneous data of the prevalence of multiple comorbidities. In our study, we systematically and rigorously assessed the comorbidities used to calculate two commonly used comorbidity indices, particularly in autoimmune diseases, making our findings relevant to presumably autoimmune-related ILD such as IPAF. Our study was the first to use comorbidity indices CCI and RDCI to assess the comorbidity burden in this specific population and demonstrate their usefulness in assessing functional and survival outcomes. Specifically, this study supports the use of RDCI in this population, a possible advantage for rheumatologists seeing these patients. Our results also emphasize the need for comprehensive evaluation of IPAF patients and multiple tools for optimal management and monitoring, including HRCT imaging, PFT monitoring, and ILD-GAP and comorbidity assessments.

We acknowledge several limitations of our study. Given the retrospective nature of the study, the variables were collected by medical record review which can lead to misclassification bias, attrition bias, and the presence of missing data. To mitigate the effects of misclassification bias of predictor variables, we used precise and consistent definitions of the key comorbidities and variables to guide the data collection by two clinical researchers (with familiarity with both the medical record and management of these conditions and comorbidities). This was a single center study so our cohort may phenotypically be different from IPAF cohorts at other center, limiting generalizability of our findings. The serological testing and rheumatological evaluation were variable in each of the patients. IPAF is inherently heterogeneous and it is possible that some patients within the cohort were more similar phenotypically to specific rheumatic diseases such as systemic lupus erythematosus (SLE) or systemic sclerosis (SSc), increasing the risk of dying from causes usually associated with those diseases (such as kidney disease in SLE or PAH in SSc). However, at the time of the IPAF cohort creation, none of the patients classified as a defined rheumatic disease based on the available data, underscoring the difficulty in assessing the prognosis and risk factors for poor outcomes in these patients. Additionally, imaging data was collected and labeled as UIP or non-UIP by an experienced ILD pulmonologist. However, loss to follow-up could have introduced selection bias. Patients who did not have repeat PFT data were excluded from the study, which could have been due to unknown death or a different cause that was not recorded in the medical record. Finally, due to the retrospective design of the study, we did not have access to the death certificates and thus were unable to determine the cause of death for the majority of the patients who died. Therefore, we were unable to confirm the significance of prevalent CVD, CVA, CHF, lymphoma, and DM as contributing factors to mortality as compared to the lung disease.

Our study was not designed to assess for the effect of treatment on the outcomes. A large proportion of patients in our study was on treatment, which varied significantly in type of medication and duration of medication administration. This limited our ability to meaningfully and effectively control for this factor. As our study is observational, we had to assume that standard of care was met for each patient. Future prospective studies may help more effectively control for medication use.

Similarly, our study was not designed to capture acute exacerbation due to the retrospective nature of the study and information being obtained from medical record review. Our results showed concordant results of CCI and RDCI being useful tools in assessing time to event using both functional decline and mortality outcomes, strengthening our confidence in the validity of the results. Finally, while our results highlight the need for comorbidity screening for patients with IPAF, we were unable to evaluate whether controlling comorbidities would mitigate the detrimental outcomes.

In summary, our study addresses an important healthcare gap for patients with IPAF, as few studies have examined comorbidities in this population and no studies have evaluated the effect of comorbidities on longitudinal outcomes in IPAF. Multiple comorbidities are highly prevalent in IPAF. Their burden, as assessed by comorbidity indices, influences functional and survival outcomes. Importantly, our study highlights the utility of two common comorbidity indices in assessing the outcomes of patients with IPAF. Particularly, the comorbidity burden in IPAF may be slightly better assessed by RDCI than CCI due to the inclusion of fracture and depression, both encountered in our cohort. Importantly, several of the comorbidities frequently seen in our cohort are modifiable and can be optimized, including DM, depression, COPD, GERD and others. Furthermore, potential mechanisms underlying CVA/CVD, CKD, and fracture with lung disease progression in IPAF need to be explored. Further prospective studies are needed to investigate whether aggressive treatment of comorbidities will mitigate poor outcomes. Clinicians taking care of patients with IPAF should be aware of common comorbid conditions, the potential prognostic implications of comorbidity indices, and the increased morbidity effect of specific comorbidities in this patient population.

## Supporting information

S1 Table Charlson Comorbidity Index (CCI). Adapted from: Charlson, et al. A new method of classifying prognostic comorbidity in longitudinal studies: development and validation. J Chronic Dis. 1987;40(5):373–83.
(DOCX)

S2 Table Rheumatic Disease Comorbidity Index (RDCI). Adapted from: England, et al. Validation of the rheumatic disease comorbidity index. Arthritis Care Res (Hoboken) (2015) 67(6):865–72.
(DOCX)

S3 Table Interstitial Lung Disease Gender-Age-Physiology (ILD-GAP) index. Adapted from: Ryerson CJ, Vittinghoff E, Ley B,et al.Predicting survival across chronic interstitial lung disease: the ILD-GAPmodel.Chest2014; 145: 723–728.
(DOCX)

S1 File Comorbidity data collection. (DOCX)

## List of abbreviations

IPAF: interstitial pneumonia with autoimmune features
ILD: interstitial lung disease
RD: rheumatic disease
CCI: Charlson Comorbidity Index
RDCI: rheumatic disease comorbidity index
GAP: index: Gender-Age-Physiology index
FVC: forced vital capacity
DLCO: diffusing capacity for carbon monoxide
IPF: idiopathic pulmonary fibrosis
UTSW: University of Texas Southwestern
ERS: European Respiratory Society
ATS: American Thoracic Society
PFT: pulmonary function tes
EMR: electronic medical record
HRCT: high-resolution computed tomography
UIP: usual interstitial pneumonia
FEV1: forced expiratory volume in one second
HR: hazard ratio
CI: confidence interval
GERD: gastroesophageal reflux disease
COPD: chronic obstructive pulmonary disease
DM: diabetes mellitus
CVA: cerebrovascular accident
CVD: cardiovascular disease
CKD: chronic kidney disease
CHF: congestive heart failure
