# Supplementary material for: Evaluation of comorbidity burden on disease progression and mortality in patients with interstitial pneumonia with autoimmune features: A retrospective cohort study
Source: PLoS One. 2025 Feb 4;20(2):e0316762. doi: 10.1371/journal.pone.0316762 (PMC11793734; doi:10.1371/journal.pone.0316762)
Supplement: S1 Table — Adapted from: Charlson, et al. A new method of classifying prognostic comorbidity in longitudinal studies: development and validation. J Chronic Dis. 1987;40(5):373–83. (DOCX) [file pone.0316762.s001.docx]

**Supplementary Table S1: Charlson Comorbidity Index (CCI)**

| *Age* | *Points* |
| --- | --- |
| <50 years  50-59 years  60-69 years  70-79 years  ≥ 80 years | 0  1  2  3  4 |
| *Comorbid Condition* |  |
| Prior myocardial infarction | 1 |
| Congestive heart failure | 1 |
| Peripheral vascular disease | 1 |
| Cerebrovascular disease | 1 |
| Dementia | 1 |
| Chronic obstructive pulmonary disease | 1 |
| Rheumatologic disease | 1 |
| Peptic ulcer disease | 1 |
| Diabetes Mellitus   - Diet-controlled - Uncomplicated - Complicated (end-organ damage) | 0  1  2 |
| Hemiplegia | 2 |
| Renal disease (moderate = creatinine >3 mg/dL or severe = on dialysis or post kidney transplant) | 2 |
| Solid tumor malignancy   - Without metastases - With metastases | 2  6 |
| Leukemia | 2 |
| Lymphoma | 2 |
| Liver disease   - Mild (chronic hepatitis or cirrhosis without portal hypertension) - Moderate to severe (cirrhosis and portal hypertension) | 1  3 |
| Acquired immunodeficiency syndrome (AIDS) | 6 |

Adapted from: Charlson, et al. A new method of classifying prognostic comorbidity in longitudinal studies: development and validation. J Chronic Dis. 1987;40(5):373-83.
