# Supplementary material for: Evaluation of comorbidity burden on disease progression and mortality in patients with interstitial pneumonia with autoimmune features: A retrospective cohort study
Source: PLoS One. 2025 Feb 4;20(2):e0316762. doi: 10.1371/journal.pone.0316762 (PMC11793734; doi:10.1371/journal.pone.0316762)
Supplement: S2 Table — Adapted from: England, et al. Validation of the rheumatic disease comorbidity index. Arthritis Care Res (Hoboken) (2015) 67(6):865–72. (DOCX) [file pone.0316762.s002.docx]

**Supplementary Table S2: Rheumatic Disease Comorbidity Index (RDCI)**

| *Comorbid Condition* | *Points* |
| --- | --- |
| Lung disease | 2 |
| Fracture | 1 |
| Depression | 1 |
| Diabetes | 1 |
| Cancer | 1 |
| Hypertension  OR  Heart Attack/Cardiovascular Disease/Stroke | 1  2 |
| Ulcer/Stomach Problem | 1 |

Adapted from: England, et al. Validation of the rheumatic disease comorbidity index. Arthritis Care Res (Hoboken) (2015) 67(6):865-72.
