# Supplementary material for: Evaluation of comorbidity burden on disease progression and mortality in patients with interstitial pneumonia with autoimmune features: A retrospective cohort study
Source: PLoS One. 2025 Feb 4;20(2):e0316762. doi: 10.1371/journal.pone.0316762 (PMC11793734; doi:10.1371/journal.pone.0316762)
Supplement: S3 Table — Adapted from: Ryerson CJ, Vittinghoff E, Ley B,et al.Predicting survival across chronic interstitial lung disease: the ILD-GAPmodel.Chest2014; 145: 723–728. (DOCX) [file pone.0316762.s003.docx]

**Supplementary Table S3: Interstitial Lung Disease Gender-Age-Physiology (ILD-GAP) Index**

|  | *Predictor* | *Points* |
| --- | --- | --- |
| ILD | ILD Subtype   - IPF - Unclassifiable ILD - RD-ILD/Idiopathic NSIP - Chronic HP (Hypersensitivity Pneumonitis) | 0  0  -2  -2 |
| G | Gender   - Female - Male | 0  1 |
| A | Age (Years)   - ≤ 60 - 61-65 - > 65 | 0  1  2 |
| P | Physiology   - FVC % predicted   > 75  50 – 75  < 50   - DLCO % predicted   > 55  36 – 55  ≤ 35  Cannot perform | 0  1  2  0  1  2  3 |

Adapted from: Ryerson CJ, Vittinghoff E, Ley B,et al.Predicting survival across chronic interstitial lung disease: the ILD-GAPmodel.Chest2014; 145: 723–728.
