# Supplementary material for: Evaluation of comorbidity burden on disease progression and mortality in patients with interstitial pneumonia with autoimmune features: A retrospective cohort study
Source: PLoS One. 2025 Feb 4;20(2):e0316762. doi: 10.1371/journal.pone.0316762 (PMC11793734; doi:10.1371/journal.pone.0316762)
Supplement: S1 File — (DOCX) [file pone.0316762.s004.docx]

**S1 File. Comorbidity Data Collection**

- Hypertension included a systolic blood pressure greater than 130 or diastolic greater than 80 on two separate visits or if listed among previous diagnoses and/or on anti-hypertensive treatment (unless the patient was using an antihypertensive agent for a documented alternative indication, such as Raynaud’s phenomenon).
- Myocardial infarction (MI) was a history of definite or probable MI with electrocardiogram or cardiac enzyme changes.
- Congestive heart failure (CHF) included a chart diagnosis of CHF or evidence of systolic or diastolic dysfunction (≥grade 2) on an echocardiogram.
- Peripheral vascular disease (PVD) included intermittent claudication, arterial insufficiency, or an untreated thoracic/abdominal aneurysm larger than 6 centimeters (cm) in diameter.
- Cerebrovascular accidents (CVA) and transient ischemic attacks (TIA) with or without residual effects were included if listed under chart diagnoses.
- Chronic obstructive pulmonary disease (COPD) included bronchitis, asthma, or chronic obstructive defects seen on PFTs.
- Obstructive sleep apnea (OSA) history was extracted from chart diagnoses or if present on sleep study results.
- Gastroesophageal reflux disease (GERD) was included if listed among chart diagnoses and based upon review of barium swallow and manometry studies.
- Ulcer disease included any chart diagnosis of peptic ulcer disease (PUD) or history of treatment for a gastrointestinal ulcer with or without bleeding.
- Liver disease included hepatitis, cirrhosis, portal hypertension, or variceal bleeding as reviewed in chart diagnoses, liver enzymes, and relevant imaging.
- Diabetes mellitus (DM) was reviewed in chart diagnoses and hemoglobin A1C levels.
- Chronic kidney disease (CKD) was evaluated based upon established diagnosis and review of recent creatinine and glomerular filtration rates (GFR) and was included if GFR was < 60 mL/min.
- Malignancy history was extracted from chart review and included solid tumors including skin cancers, leukemia, and lymphoma; metastatic status was recorded.
- Acquired immunodeficiency syndrome (AIDS) and human immunodeficiency virus (HIV) infection were included based on chart diagnosis and review of infectious disease labs.
- Hemiplegia was defined as a chart diagnosis of unilateral paralysis.
- Fractures of the hip, spine, or leg were included if present in the patient’s history or on available imaging.
- Osteoporosis was present if present on bone density scan or if previously diagnosed and patient was on bisphosphonate or alternative osteoporosis treatment.
- Depression was considered present if a chart diagnosis of depression or an anti-depressant was listed in the medication list (unless the anti-depressant medication was used for an alternative documented indication such as anxiety).
- Dementia and cognitive impairment were included if present in chart diagnoses.
- Hypothyroidism was considered present if recorded in chart diagnoses or history or if patient was on thyroid hormone supplementation such as levothyroxine.
